# The Use of Quantitative Electroencephalography (QEEG) to Assess Post-COVID-19 Concentration Disorders in Professional Pilots: An Initial Concept

**DOI:** 10.3390/brainsci13091264

**Published:** 2023-08-30

**Authors:** Marta Kopańska, Łukasz Rydzik, Joanna Błajda, Izabela Sarzyńska, Katarzyna Jachymek, Tomasz Pałka, Tadeusz Ambroży, Jacek Szczygielski

**Affiliations:** 1 Department of Pathophysiology, University of Rzeszow, 35-959 Rzeszow, Poland; 2 Institute of Sports Sciences, University of Physical Education, 31-571 Kraków, Polandtadek@ambrozy.pl (T.A.); 3 Institute of Health Sciences, Medical College, University of Rzeszow, Kopisto 2a, 35-959 Rzeszow, Poland; joanna.blajda@gmail.com; 4 Students Science Club “Reh-Tech”, Institute of Medical Sciences, University of Rzeszow, 35-959 Rzeszow, Poland; 5 Department of Physiology and Biochemistry, Faculty of Physical Education and Sport, University of Physical Education, 31-571 Kraków, Poland; 6 Faculty of Medicine, University of Rzeszow, 35-959 Rzeszow, Poland; 7 Department of Neurosurgery, Faculty of Medicine, Saarland University, 66421 Homburg, Germany

**Keywords:** quantitative EEG analysis, QEEG, brain, pilots, concentration, COVID-19

## Abstract

Announced by WHO in 2020, the global COVID-19 pandemic caused by SARS-CoV-2 has affected many people, leading to serious health consequences. These consequences are observed in the daily lives of infected patients as various dysfunctions and limitations. More and more people are suffering post-COVID-19 complications that interfere with or completely prevent them from working or even functioning independently on a daily basis. The aim of our study was to demonstrate that innovative quantitative electroencephalography (QEEG) can be used to assess cognitive function disorders reported after the COVID-19 pandemic. It is worth noting that no similar study has been conducted to date in a group of pilots. The QEEG method we used is currently one of the basic neurological examinations, enabling easy observation of post-COVID-19 changes in the nervous system. With the innovativeness of this technique, our study shows that the use of quantitative electroencephalography can be a precursor in identifying complications associated with cognitive function disorders after COVID-19. Our study was conducted on twelve 26-year-old pilots. All participants had attended the same flight academy and had contracted SARS-CoV-2 infection. The pilots began to suspect COVID-19 infection when they developed typical symptoms such as loss of smell and taste, respiratory problems, and rapid fatigue. Quantitative electroencephalography (QEEG), which is one of the most innovative forms of diagnostics, was used to diagnose the patients. Comparison of the results between the study and control groups showed significantly higher values of all measurements of alpha, theta, and beta2 waves in the study group. In the case of the sensorimotor rhythm (SMR), the measurement results were significantly higher in the control group compared to the study group. Our study, conducted on pilots who had recovered from COVID-19, showed changes in the amplitudes of brain waves associated with relaxation and concentration. The results confirmed the issues reported by pilots as evidenced by the increased amplitudes of alfa, theta, and beta2 waves. It should be emphasized that the modern diagnostic method (QEEG) presented here has significant importance in the medical diagnosis of various symptoms and observation of treatment effects in individuals who have contracted the SARS-CoV-2 virus. The present study demonstrated an innovative approach to the diagnosis of neurological complications after COVID-19.

## 1. Introduction

On 11 March 2020, COVID-19 was declared a global pandemic by the WHO. The pandemic has affected many people, leading to various serious health consequences. Thus far, the rapid spread of SARS-CoV-2 has caused significant harm to public health and the economy [1,2,3,4]. It has also led to serious economic consequences due to disruptions in occupational functioning [5,6]. Infected patients exhibit symptoms of severe respiratory tract infections, which can lead to acute respiratory distress syndrome (ARDS) and multiorgan complications [7]. Patients with COVID-19 also exhibit neurological symptoms such as headaches, dizziness, muscle pain, loss of smell, and encephalopathy [8,9,10,11]. These disorders significantly impact lifestyle and lead to problems with performing daily activities [11]. In our study, we used a quantitative electroencephalogram as an innovative diagnostic tool. The method was chosen because it is a relatively easy-to-use and effective method among many functional brain imaging techniques that provide high reliability and allow for low-cost research, which is an additional advantage [12]. QEEG involves quantitative analysis of EEG recordings using statistical signal processing [13]. The use of quantitative electroencephalography instead of the usual EEG allows for an easier analysis of the results due to their better visualization [14]. It is also worth noting that quantitative electroencephalography is a very sensitive examination and can detect even very small changes in the functioning of the cerebral cortex [15]. It is also a reliable and non-invasive diagnostic tool for the quantitative determination of cortical synaptic damage or loss in the clinical assessment of neurodegenerative diseases [16]. Accurate analysis of QEEG results enables planning of the appropriate EEG biofeedback therapy [17], which is an effective method for reducing stress and supporting self-regulation [18,19]. Compared to conventional EEG, the QEEG method provides a more accurate assessment of brain function, making it useful for identifying minimal changes in brain wave activity [20]. In the present study, we decided to use QEEG to evaluate the quantitative distribution of brain waves after COVID-19 in professional pilots complaining of concentration problems and discomfort after coronavirus infection. The method we chose will help us accurately assess brain function and thoroughly diagnose disorders occurring in the CNS. It is worth noting that the profession of a pilot requires high resilience to stressful situations, as this work involves making quick decisions and analyzing large amounts of data [21,22]. Given the before-mentioned scientific reports indicating the negative effects of SARS-CoV-2 infection on the nervous system, it can be concluded that our examination, which was based on the analysis of brain waves in post-COVID-19 pilots conducted using a modern method of quantitative electroencephalography, may become a breakthrough in the diagnosis of post-COVID-19 cognitive disorders. A very small number of similar studies have been conducted to date to analyze QEEG results in post-COVID-19 cognitive disorders in different groups.

## 2. Material and Methods

### 2.1. Participants

The study was conducted on twelve 26-year-old pilots. All participants attended the same flight academy and had frequent interactions with each other. As a result, they contracted SARS-CoV-2. The pilots began to suspect COVID-19 when they developed typical symptoms such as loss of smell and taste, respiratory problems, and rapid fatigue. They all underwent PCR tests and all tested positive. The pilots were isolated and underwent a fourteen-day quarantine. They did not need outpatient treatment and contacted their family physicians only by phone. The pilots underwent medical examinations by their general practitioners, and parameters of inflammatory state (D-dimers, CRP, blood morphology) were evaluated. Importantly, none of the study participants had been vaccinated against COVID-19, which standardized the study group. Patients were taking lung diastolic medications due to nagging mild dyspnea. In their medical history, patients reported excessive fatigue. They described it as something that had never happened to them before. All participants experienced similar symptoms of COVID-19. In general, the profession of a pilot is associated with the requirement of having excellent health. The study group also reported worse results in the above-mentioned tests, which occurred after recovering from COVID-19. During this time, all participants also noticed concentration problems, which intensified after the illness. Therefore, the pilots underwent routine and regular examinations to assess their psychomotor health and ability to perform the work of a pilot. Fatigue was chronic in nature and exacerbated when performing any physical activity, even such as going to the toilet. When performing more strenuous activities, patients reported being unable to take a deep breath. After quarantine, pilots were retested with a PCR test for the virus. The results were negative. Although the pilots returned to their work activities, they reported a lack of concentration and focus, irritability, and disorientation when receiving large amounts of stimuli. Furthermore, they noticed a deterioration in their mental health status. While standing in the cockpit, they were receiving so many visual and auditory inputs from the control panel that they were unable to focus on their activities. The patients were referred for follow-up examinations, during which it was decided to perform QEEG. The characteristics of the study group are presented in Table 1.

The results of the QEEG examinations conducted on a group of 12 pilots were compared to a control group consisting of 8 pilots from the same flight academy who did not report any concentration problems, additional medical conditions, visual impairments, or diagnosed dysfunctions. In the GSES tests and psychological evaluation, all parameters in the control group were within the normal range. It is worth noting that the selected control group consisted of different members than the study group because we were not able to predict the dynamically developing situation of the number of people infected with SARS-CoV-2 and the complications that would occur after the disease. Currently, many studies analyzing post-COVID-19 complications use different control groups than the study groups. It is also worth noting that the individuals in the control group contracted COVID-19 in a very similar way but did not experience any complications. These individuals were also not vaccinated against COVID-19, similar to the study group. The study was conducted in accordance with the Declaration of Helsinki, and it was approved by the Ethics Committee of the University of Rzeszow (protocol code 2022/038 from 6 April 2022).

### 2.2. QEEG Analysis

QEEG analysis was performed in a relaxed state and with open eyes. A biofeedback showing brain-wave frequency was obtained. During the QEEG examination, values are read from all points (Cz, C4, C3, Fz, F3, F4, P3, and P4). The EEG signal was recorded using wet electrodes. The study analyzed alpha, theta, delta, SMR, beta1, and beta2 waves. We took into account the theta, SMR, and beta2 µV amplitude (the most important wave for concentration) from all points in which there were significant changes. Due to the disturbing symptoms, the pilots decided to retest themselves using psychological testing for pilot assessment. These tests are designed to assess the aptitude of a pilot. Individual psychomotor test scores declined from the results of the tests taken before the illness and COVID-19 pandemic. After patients were referred to us, we administered a standard GSES questionnaire. This is a scale to assess general self-efficacy. It assesses a person’s belief in dealing with difficult situations and obstacles. This questionnaire consists of 10 theses to which the patient must respond by choosing one of four possible answers. The resulting score is then summed up, ranging from 10 to 40 points. The lower the score, the lower the sense of self-efficacy. The following are the scores obtained from the pilots (Table 2).

It can be seen from the results presented that the pilots presented a low to medium level of self-efficacy, which has a negative effect on performing their work.

### 2.3. Statistical Analysis

Distributions of amplitudes were analyzed using means, standard deviations, medians, and quartiles. The Mann–Whitney test was used to compare amplitudes between the two groups. The significance level for all statistical tests was set at 0.05. R 4.3.0. software was used for computations [23].

## 3. Results

### 3.1. Theta Waves

**Figure 1:**
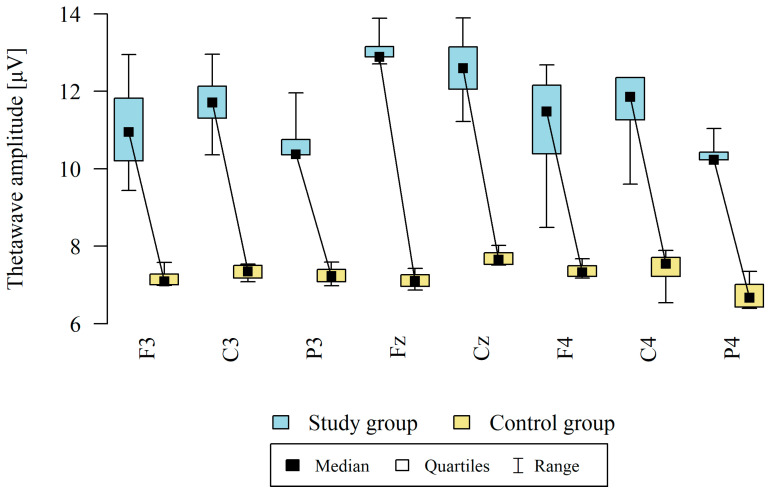
Comparison of theta wave amplitudes in the frontal (Fz, F3, F4), central (Cz, C3, C4), and occipital (P3, P4) points between the study group (*n* = 12) and the control group (*n* = 8). The comparison of the values of quantitative variables in the two groups was performed using the Mann–Whitney test (*p* < 0.05).

Values of *p* < 0.05 indicate statistically significant differences:

The amplitude at each point was significantly higher in the study group (Table 3, Figure 1).

### 3.2. Sensorimotor Waves (SMR)

**Figure 2:**
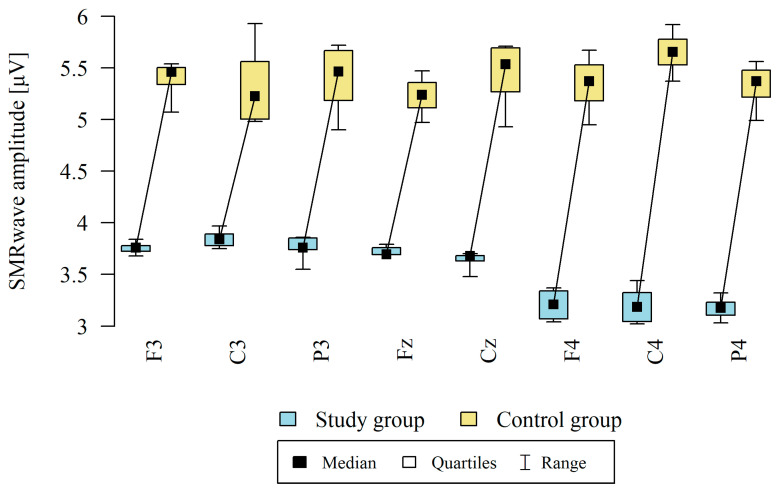
Comparison of SMR wave amplitudes in the frontal (Fz, F3, F4), central (Cz, C3, C4), and occipital (P3, P4) points between the study group (*n* = 12) and the control group (*n* = 8). The comparison of the values of quantitative variables in the two groups was performed using the Mann–Whitney test (*p* < 0.05).

Values of *p* < 0.05 indicate statistically significant differences:

The amplitude at each point was significantly higher in the control group (Table 4, Figure 2).

### 3.3. Beta2 Waves

**Figure 3:**
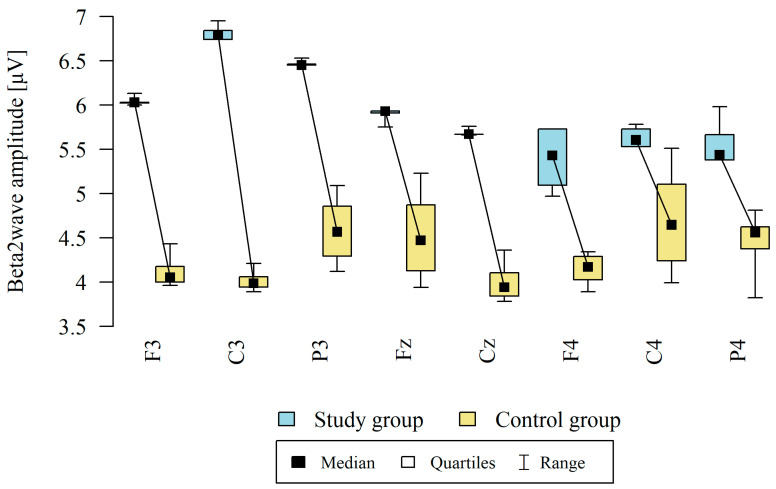
Comparison of beta 2 wave amplitudes in the frontal (Fz, F3, F4), central (Cz, C3, C4), and occipital (P3, P4) points between the study group (*n* = 12) and the control group (*n* = 8). The comparison of the values of quantitative variables in the two groups was performed using the Mann–Whitney test (*p* < 0.05).

Values of *p* < 0.05 indicate statistically significant differences:

The amplitude at each point was significantly higher in the study group (Table 5, Figure 3).

### 3.4. Alpha Waves

**Figure 4:**
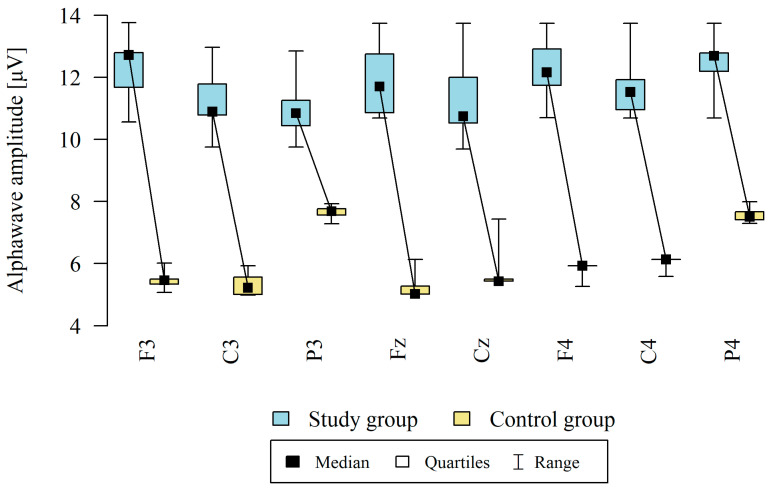
Comparison of alpha wave amplitudes in the frontal (Fz, F3, F4), central (Cz, C3, C4), and occipital (P3, P4) points between the study group (*n* = 12) and the control group (*n* = 8). The comparison of the values of quantitative variables in the two groups was performed using the Mann–Whitney test (*p* < 0.05).

Values of *p* < 0.05 indicate statistically significant differences:

The amplitude at each point was significantly higher in the study group (Table 6, Figure 4).

## 4. Discussion

The aim of our study was to develop a brainwave model using quantitative electroencephalography in professional pilots reporting problems with concentration and other cognitive disorders who had contracted SARS-CoV-2. The main component of our study was to evaluate changes in alpha, theta, beta2, and SMR wave amplitudes compared to QEEG parameters in the control group. No study to date has become a valid diagnostic criterion for studying the disorders occurring in the central nervous system after infection with SARS-CoV-2. Therefore, the use of quantitative electroencephalography in our study is a new research perspective.

Although COVID-19 primarily affects the lungs, it can also generate debilitating conditions that affect many systems, including the central nervous system (CNS) [2,24]. The SARS-CoV-2 virus activates mechanisms in the body that involve pro-inflammatory cytokine pathways and oxidative stress, which can lead to an imbalance in glutamate regulation. This underlies the development of mental disorders [25]. The consequences of infection with SARS-CoV-2 include symptoms of chronic fatigue, depression, and mental health disorders, as well as cognitive disorders such as brain fog and difficulties in concentration and memory [26,27,28]. Neurological symptoms persist for a long time after recovery from the acute phase of infection with the virus, often referred to as “long COVID” or the “long tail of COVID-19” [29], and this was also observed in our patients.

The quantitative electroencephalography we used is an increasingly popular method for the diagnosis of neurological disorders [30]. It helps assess the functioning of the central nervous system, especially in the cognitive aspects [31]. QEEG allows for the analysis of brain activity, but it also demonstrates the interaction between different areas of the cerebral cortex. Moreover, the use of this method in a study of individuals who contracted SARS-CoV-2 is a very interesting reference, as QEEG analysis can help indicate the relationship between post-COVID cognitive disorders and SARS-CoV-2 infection. Quantitative electroencephalography (QEEG) is related to numerical analysis and refers to visual transformations of unprocessed EEG signals, which facilitates the analysis of the results [32]. EEG records the signal using wet electrodes. The application of wet electrodes in electroencephalography (EEG) requires a conductive solution to carry the electrolyte between the electrode and the skin of the head [33]. A low-impedance wet electrode ensures good signal quality [34]. Thompson et al. noted that quantitative analysis is more accurate in the assessment of the current state of the brain and identifying problems compared to a conventional electroencephalographic examination. QEEG enables the determination of the underlying abnormalities in the functioning of cerebral cortical areas and the correlation of the clinical state with power maps and QEEG charts [35]. It is worth noting that more and more authors are also focusing on the use of quantitative electroencephalography in diagnosing disorders related to cognitive functions due to the accuracy of this method [36]. This makes it possible to conclude that the QEEG method is one of the most reliable methods for diagnosing cognitive disorders.

Before we move on to the analysis of the results, it is worth noting that the present research findings should be interpreted with caution, as we are not able to verify whether the main cause of the symptoms in pilots was the SARS-CoV-2 virus. After analyzing the available research, we have noticed that, so far, no studies have been conducted using quantitative electroencephalography on a group of pilots. However, it should be noted that several studies have already been conducted on this professional group using electroencephalography, but from a different perspective and unrelated to COVID-19. The only study that demonstrates the impact of COVID-19 on this professional group relates to the risk of SARS-CoV-2 virus infection in small aircraft, which can occur during flights between pilots [37]. Therefore, in our opinion, the use of quantitative electroencephalography as a method to diagnose the impact of the SARS-CoV-2 virus on the central nervous system of professional pilots represents an innovative approach, as it is the first study conducted on this professional group in which cognitive function disorders have appeared after recovering from the coronavirus [38,39,40,41,42,43,44,45,46,47,48,49,50,51,52,53].

Analysis of the results showed repeated phenomena in all patients: a significant increase in theta wave amplitude, a decrease in SMR waves, and an increase in alpha and beta2 waves compared to the control group. In our study, unfavorable amplitudes of alpha, theta, beta2, and SMR waves are presented in tables showing measurements at the F3, C3, P3, Fz, Cz, F4, C4, and P4 points for both the study and control groups. Analysis of the results revealed significant differences in the amplitudes of the tested frequencies in the post-COVID-19 group compared to the controls. Increased values of beta2, alpha, and theta waves and decreased values of SMR waves in the study group were observed for all points tested. Similar results were obtained in a study conducted by Kopańska et.al. in 2022, who also used innovative quantitative electroencephalography. The researchers observed an increase in alpha, theta, and beta2 frequencies and a decrease in SMR frequency. The examinations were conducted on employees of the University of Rzeszow who complained of the aforementioned symptoms after being infected with SARS-CoV-2. Interestingly, a few months before the outbreak of the pandemic, these individuals participated in a QEEG screening to assess their brain activity. The study participants showed an increase in theta, alpha, and SMR frequencies in the right hemisphere, an increase in beta2 amplitude compared to SMR in both hemispheres, an increase in beta1 in the left hemisphere, and a decrease in SMR values [54]. The study confirmed the increased alpha, theta, and beta2 values and decreased sensorimotor rhythm in patients complaining of post-COVID-19 cognitive disorders. This leads to the conclusion that COVID-19 had a negative effect on the central nervous system, as it significantly affects the amplitude of the brain waves analyzed.

In the study we presented, high beta2 (18–30 Hz) wave values were observed for every lead used. These waves are produced in the brainstem and cerebral cortex [35]. In our patients, the wave-related values exceeded the reference levels and were significantly higher than the values documented in the control group. An elevated beta2-wave state is perceived as unfavorable as it is associated with high emotional tension [55]. The resulting increased beta2 waves in both hemispheres indicate increased levels of stress and emotional tension, which was the case in our patients. Often, these waves are responsible for the release of adrenaline, and remaining in such a state for a longer time results in frequent fatigue [56]. This wave reproduces brain activity, which is correlated with wakefulness [57]. In the pilot group studied, high beta2 wave amplitudes can be interpreted as unfavorable. They may be due to numerous stressful situations and a state of tension associated with constant decision-making. It is also worth noting that the elevated beta2-wave state in the pilots studied may also occur because of the intense focus during the flight. Despite the reasons presented above for the presence of elevated beta2 wave amplitudes in the study group, it should not be ruled out that the main cause may be the previous infection with SARS-CoV-2. These speculations arise from the fact that the control group was also a group of pilots, but they had never been infected with the virus. The results obtained in both groups showed significant differences in brain wave activity in each of the measured leads, with increased beta2 wave activity observed in pilots after coronavirus infection.

In the frequency range of sensorimotor waves, a reduction in amplitude was observed compared to the control group in both hemispheres. Sensorimotor rhythm (SMR) is a type of brain wave that occurs in the frequency range of 12–15 Hz. These waves originate from the ventral basal nucleus of the thalamus. SMR rhythms are formed at rest and with a low concentration of attention on sensory inputs and low motor activity [35]. The rhythmic activity associated with motion detection is related to a “relaxed” mental state [58]. It occurs mainly in the sensory and motor regions of the brain and is associated with the activation of the motor cortex. Suppression of SMR activity can interfere with the acquisition, perception, and processing of information [59]. It is also believed that excessively low SMR wave frequencies accompany attention deficits [54], which was observed in our patients after comparing the obtained results with the values obtained in the control group. In the group of patients studied, who were post-COVID-19 pilots, the low frequencies of SMR waves can be attributable to SARS-CoV-2 virus infection. In many publications, authors have analyzed brain fog, which is characterized by a low level of attention and is considered one of the many complications experienced by patients after COVID-19 [60].

Another wave with increased amplitudes was the alpha wave. If alpha waves are too high, they can cause anxiety, indicating that the patient is tense, which manifests itself in difficulty concentrating and a general reduction in the level of cognitive function activity [61], which was the case in the patients we analyzed. The alpha rhythm (8–12 Hz) occurs in states of relaxation and wakefulness, with the eyes closed, and is blocked when the eyes are opened [62]. It is most easily observed in the posterior leads [35]. Alpha waves that are too high can cause attention problems and fatigue. In the case of the patients we analyzed, who had been infected with SARS-CoV-2, the increased values of alpha-wave amplitudes compared to the control group may be indicative of the concentration problems reported by the participants, which may be a consequence of COVID-19, as confirmed by many authors studying the post-COVID-19 complications. Additionally, the control group consisted of pilots of the same age who had never been infected with the SARS-CoV-2 virus [63,64].

Theta waves occur in the frequency range of around 4–8 Hz. The waves are generated in the thalamus and limbic system. The theta wave is associated with the ability to control responses to stimuli and retrieve information from memory. Creative thinking is also associated with theta rhythm [35]. The high amplitude of theta waves can also be associated with various emotional states, such as severe nervousness and disquietude, which can lead to feeling distracted and attention problems [55,65,66]. The elevated theta waves observed in our patients also confirm the symptoms reported by pilots who reported experiencing symptoms of attentional distraction, concentration difficulties, and anxiety during flight. These symptoms emerged after contracting SARS-CoV-2, which may be related not only to the infection itself but also to the stress response caused by the deterioration of their health and the fear of complications from the disease.

It can be concluded that the waveform values show a correlation between the results obtained and the post-COVID-19 disorders reported by pilots.

In another pilot research, the authors also performed electroencephalography to confirm neurological symptoms after COVID-19, which underscores the importance of this test in the diagnosis of post-COVID-19 complications occurring in the CNS.

Keith J. Kincaid and his colleagues presented a 71-year-old patient with post-COVID-19 neurological disorders of unknown origin, observed in computed tomography, magnetic resonance imaging, and EEG. At the time of the occurrence of the patient’s symptoms, COVID-19 was not detected, leading the authors to suggest that many neurological disorders may develop in patients after COVID-19 [67]. In an EEG study conducted by M. Flamand et al., an unfavorable association between the occurrence of neurological symptoms and COVID-19 was also observed. In an 80-year-old woman, a progressive neurological process was observed due to a remarkable periodic feature of triphasic waves in the EEG [68]. Furthermore, Giordano Cecchetti and his co-authors conducted EEG tests on patients complaining of memory and cognitive problems after COVID-19. After conducting the examination and analyzing the delta frequency band, higher CSD was observed in the bilateral frontotemporal regions [69]. Currently, many studies focus on changes in electroencephalographic patterns in people complaining of post-COVID-19 cognitive function disorders, as an increasing number of people report problems with memory, cognitive function, and concentration. In a study by Giovanni Furlanis, which was conducted on patients complaining of cognitive impairment, altered EEG traces were observed, prevailing in the frontal regions [70]. In his study, SA Gulyaev also focused on the problem of cognitive dysfunction after COVID-19, and he conducted an electroencephalographic examination of 38 people who returned to work after recovering from the disease. The study showed that people who had new coronavirus infections for a longer time (up to 6 months) developed cognitive disorders that made it difficult for them to return to professional performance, which was confirmed by the changes found in brain bioelectrical activity. Interestingly, the author named these dysfunctions the “post-COVID syndrome” [71]. In an EEG study conducted by Jesús Pastor et al., 20 patients who had recovered from COVID-19 were found to have developed encephalopathy characterized by impaired cognitive function and altered mental status. Excessive delta activity and lower alpha and beta values were found in the study [72]. Other authors have also noted that the SARS-CoV-2 virus had a negative impact on the CNS. In an article by Dr. Hervé Vespignani et al., an EEG examination was conducted on 26 patients with severe COVID-19 who were hospitalized in several intensive care units in Paris. In five patients, a high amplitude of delta waves with no elliptical activity was observed, which was associated with cognitive deficits [73]. Furthermore, in a study by Maria Rubenga et al., it was observed that COVID-19 had an unfavorable impact on cognitive functions. After conducting an EEG study during sleep in 33 patients, a psychoaffective character of impairment of these functions was observed, which manifested in a decrease in executive functions and disturbances in memory [74]. A systematic review conducted by Katrina T. Roberto et al., analyzed 177 COVID-19 patients who reported altered mental states and other neurological symptoms after COVID-19. After analyzing the results, the authors concluded that patients with COVID-19 mostly exhibited abnormal EEG readings, which confirms that the SARS-CoV-2 virus has a negative impact on the functioning of the central nervous system [75]. Based on a review of various studies that have used electroencephalography to study post-COVID-19 cognitive disorders, it can be concluded that the concepts we proposed here are accurate. COVID-19 negatively affects the central nervous system, causing cognitive disorders, and the use of EEG to diagnose these disorders is a good diagnostic tool. We did not find studies in which researchers used an innovative QEEG test to diagnose cognitive disorders after COVID-19. Therefore, our study demonstrates a novel diagnostic approach to assessing such problems.

Our results suggest that the coronavirus has a negative impact on the central nervous system, as it significantly affects the amplitude of the analyzed brain waves compared to the frequencies of the waves in the control group. The obtained results of the electroencephalographic examination in pilots indicate significant changes in the amplitude of emitted brain waves, associated with concentration and a state of calmness, confirming the complaints reported by pilots that occurred after contracting COVID-19. Since the study was conducted after infection with SARS-CoV-2, it can be concluded that the coronavirus has a negative effect on brain activity, causing concentration disorders manifested by dispersion, disorientation, and irritability [76]. The association of SARS-CoV-2 with acute and chronic neurological symptoms is the subject of many current studies investigating possible direct and indirect viral infection of the nervous system [1,77,78]. Our assumptions that COVID-19 negatively affects cognitive function are potentially true, as we see that a significant number of studies have described cases of the negative impact of the virus on cognitive function. In an article aimed at defining the concept of long COVID, Nisreen A. Alwan and Luke Johnson stated that infection with SARS-CoV-2 carries long-term consequences, such as persistent fatigue, shortness of breath, headaches, chest tightness, muscle pain, and heart palpitations. Post-COVID symptoms involve multiple systems and have a broad range, typically with a variable or recurrent character. In a large percentage of people who failed to fully recover, cognitive problems also appeared, including poor memory, poor concentration and brain fog [79].

In a study by Bram van den Borst et al., the health status of 124 patients was evaluated three months after recovery from acute coronavirus disease. To assess cognitive function accurately, all patients completed questionnaires about their mental health, cognitive function, health status, and quality of life (QoL). After conducting the tests, it was observed that about one-third of patients had abnormal results related to mental health or cognitive function [80].

In one study, Riikka E. Pihlaja et al. aimed to describe the prevalence of subjective and objective cognitive dysfunction three and six months after COVID-19. The study was conducted on 184 patients, of whom one-third reported a high level of cognitive dysfunction. Cognitive function was assessed using the AB Neuropsychological Assessment Schedule (ABNAS) questionnaire and the Montreal Cognitive Assessment (MoCA). The results suggested that the problems reported by patients were subjective perceptions [81]. K.W. Miskowiak et al. also observed similar cognitive disorders in their study, with half of 194 patients after acute COVID-19 showing impaired global measures of cognitive function, memory, executive function, and verbal learning based on cognitive screening and questionnaires on subjective cognition, work functioning, and quality of life. Analysis of the results obtained in a study by K.W. Miskowiak et al. revealed impairment of global measures of cognitive function, memory, executive function, and verbal learning in patients after infection with SARS-CoV-2 [82].

In a study by Marcel S. Woo et al., an unfavorable impact of the SARS-CoV-2 virus on the central nervous system was also observed. Screening tests were conducted on young patients complaining of concentration deficits, short-term memory loss, and fatigue after recovering from COVID-19. It was found that young patients who recovered from uncomplicated COVID-19 may have lasting neuropsychological deficits [76]. Other authors also found that COVID-19 results in memory and concentration deficits. Giordano Cecchetti et al. used structured neuropsychological assessment and resting-state EEG to identify cognitive impairments in more than 50% of patients [65].

In our experiment, we also used the General Self-Efficacy Scale (GSES), which consists of 10 questions aimed at measuring the strength of the team’s general position on coping with difficult situations and obstacles. Self-efficacy is often referred to as “belief”, or the degree of confidence in one’s ability to successfully perform a specific task. The maximum score is 40, and each question is rated on a four-point scale of 1—not at all, 2—hardly, 3—moderately, and 4—exactly [54]. Our analysis of the results of the GSES test on pilots showed an average sense of self-efficacy in coping with stressful situations, as the test results ranged from 19 (the lowest score) to 27 (the highest score). The test showed that the pilots had a relatively low sense of self-worth, which also translated into the results of the electroencephalographic study we conducted [83].

So far, most studies on the impact of COVID-19 on the central nervous system have only provided a basis for further observations. Our study, which focused on the impact of SARS-CoV-2 infection on cognitive functions, is the first study conducted on both a tested group and a control group consisting of pilots. Nevertheless, it can be concluded that our findings and studies conducted by other authors suggest a negative impact of the SARS-CoV-2 virus on brain functioning, which may ultimately lead to cognitive dysfunction. Although our study was conducted on a small group of people, it can serve as a reference for other researchers due to the homogeneity of the profession and age of the study group. It also indicates that the use of quantitative electroencephalography in the assessment of post-COVID cognitive disorders is an innovative approach that may become one of the basic diagnostic tests to assess CNS disorders.

## 5. Limitations

Our study has some limitations. One of the biggest limitations is the fact that the study was conducted on a small group of people, which, in effect, does not conclusively resolve the problem presented and becomes a kind of introduction to further research. Another limitation is that, despite our efforts, we cannot access the raw EEG data because the manufacturer of the device on which we performed the tests does not disseminate the above data. Another limitation is the lack of access to data presenting the results of performance tests conducted on pilots. However, analysis of these data is not the main focus of our study, as we intended to show the brain wave activity in post-COVID-19 patients. In this study, apart from QEEG-biofeedback and the GSES scale, we did not include the MMSE scale. However, we will use it in the future when we expand our study, which, at this stage, is aimed to show the effects that infection with SARS-CoV-2 can have on the CNS, mainly leading to cognitive impairment. In the future, we would also like to focus on using similar data obtained in post-COVID-19 patients using the EEG method to determine a form of the electroencephalographic test. It is worth noting that the participants were in the same age range, attended the same aviation academy, and had similar COVID-19 symptoms, making our results more reliable.

## 6. Conclusions

Our study, which was conducted on pilots, is the first study to demonstrate the importance of quantitative electroencephalography (QEEG) in observing cognitive function disorders reported by a group of pilots who had recovered from COVID-19, and it demonstrated the vital importance of the QEEG method in the diagnostic identification of post-COVID-19 cognitive function disorders. Our study showed changes in the amplitude of brain waves associated with relaxation and concentration. The results confirmed the issues reported by pilots, as evidenced by significantly increased theta, alpha, and beta2 wave amplitudes in the study group compared to the control group. The statistical analysis showed statistically significant differences between the two groups. Although the study was conducted on a small group of people, it can be assumed that the SARS-CoV-2 virus has a negative effect on brain function, which has been confirmed by numerous previous studies. The GSES test confirmed our findings in the electroencephalographic study, as it revealed low self-efficacy in coping with stressful situations among pilots. Our study has some limitations, such as the small number of participants However, the results can inspire other researchers to conduct further studies on larger groups of individuals. It is worth noting that, at this stage, our study is aimed to signal the effects that SARS-CoV-2 can have on the CNS, leading to cognitive impairment. In the future, we would like to expand the research; by conducting many similar studies in the future, we will be able to compare and accurately identify factors in the epidemiology of the COVID-19 pandemic.

## Figures and Tables

**Table 1:** Characteristics of study participants.

	Mean	SD	Min	Max
Age	27.00	1.11	26.00	29.00
Body Height	183.75	6.60	176.00	191.00
Body Mass	87.00	3.91	83.00	92.00

SD—standard deviation, Min—minimum, Max—maximum.

**Table 2:** GSES test results.

Pilot Number	Score/Result
1	24
2	19
3	23
4	20
5	22
6	18
7	25
8	21
9	23
10	19
11	25
12	22

**Table 3:** The results of the theta waves examination (waves from Fz, F3, F4, Cz, C3, C4, P3, and P4 channels). The values express wave amplitude (in µV) with main distribution parameters (including median and quartiles).

Point	Group	N	Mean	SD	Median	Min	Max	Q1	Q3	*p*
F3	Study group	12	11.07	1.35	10.95	9.44	12.95	10.20	11.82	*p* < 0.001 *
	Control group	8	7.19	0.25	7.10	6.99	7.58	7.01	7.28	
C3	Study group	12	11.72	0.92	11.71	10.36	12.96	11.31	12.13	*p* < 0.001 *
	Control group	8	7.33	0.20	7.35	7.08	7.54	7.18	7.50	
P3	Study group	12	10.76	0.70	10.38	10.36	11.96	10.36	10.76	*p* < 0.001 *
	Control group	8	7.26	0.25	7.23	6.98	7.59	7.08	7.40	
Fz	Study group	12	13.13	0.46	12.89	12.71	13.89	12.89	13.16	*p* < 0.001 *
	Control group	8	7.12	0.23	7.10	6.87	7.43	6.96	7.26	
Cz	Study group	12	12.59	1.00	12.60	11.22	13.90	12.06	13.15	*p* < 0.001 *
	Control group	8	7.71	0.22	7.65	7.51	8.02	7.53	7.83	
F4	Study group	12	11.05	1.63	11.48	8.49	12.68	10.38	12.15	*p* < 0.001 *
	Control group	8	7.38	0.21	7.33	7.18	7.68	7.22	7.49	
C4	Study group	12	11.53	1.04	11.86	9.60	12.36	11.26	12.36	*p* < 0.001 *
	Control group	8	7.38	0.55	7.55	6.54	7.89	7.22	7.71	
P4	Study group	12	10.43	0.37	10.23	10.23	11.04	10.23	10.43	*p* < 0.001 *
	Control group	8	6.77	0.41	6.67	6.40	7.35	6.43	7.01	

*p*—Mann–Whitney test, SD—standard deviation, Q1—lower quartile, Q3—upper quartile. * statistically significant (*p* < 0.05).

**Table 4:** The results of the SMR waves examination (waves from Fz, F3, F4, Cz, C3, C4, P3, and P4 channels). The values express wave amplitude (in µV) with main distribution parameters (including median and quartiles).

Point	Group	N	Mean	SD	Median	Min	Max	Q1	Q3	*p*
F3	Study group	12	3.76	0.06	3.76	3.68	3.84	3.72	3.78	*p* < 0.001 *
	Control group	8	5.38	0.20	5.46	5.07	5.54	5.34	5.50	
C3	Study group	12	3.84	0.08	3.84	3.75	3.97	3.78	3.89	*p* < 0.001 *
	Control group	8	5.34	0.41	5.22	4.98	5.93	5.00	5.56	
P3	Study group	12	3.78	0.09	3.76	3.55	3.86	3.74	3.85	*p* < 0.001 *
	Control group	8	5.39	0.35	5.46	4.90	5.72	5.19	5.67	
Fz	Study group	12	3.72	0.04	3.70	3.69	3.79	3.69	3.76	*p* < 0.001 *
	Control group	8	5.23	0.20	5.24	4.97	5.47	5.11	5.36	
Cz	Study group	12	3.63	0.09	3.68	3.48	3.70	3.63	3.68	*p* < 0.001 *
	Control group	8	5.43	0.34	5.54	4.93	5.71	5.27	5.70	
F4	Study group	12	3.20	0.15	3.21	3.04	3.37	3.07	3.34	*p* < 0.001 *
	Control group	8	5.34	0.29	5.37	4.95	5.67	5.18	5.53	
C4	Study group	12	3.20	0.18	3.18	3.02	3.44	3.04	3.32	*p* < 0.001 *
	Control group	8	5.65	0.22	5.66	5.37	5.92	5.53	5.78	
P4	Study group	12	3.16	0.10	3.17	3.03	3.32	3.10	3.23	*p* < 0.001 *
	Control group	8	5.32	0.23	5.37	4.99	5.56	5.22	5.48	

*p*—Mann–Whitney test, SD—standard deviation, Q1—lower quartile, Q3—upper quartile. * statistically significant (*p* < 0.05).

**Table 5:** The results of the beta2 waves examination (waves from Fz, F3, F4, Cz, C3, C4, P3, and P4 channels). The values express wave amplitude (in µV) with main distribution parameters (including median and quartiles).

Point	Group	N	Mean	SD	Median	Min	Max	Q1	Q3	*p*
F3	Study group	12	6.03	0.03	6.03	6.00	6.13	6.02	6.03	*p* < 0.001 *
	Control group	8	4.12	0.20	4.05	3.96	4.43	4.00	4.18	
C3	Study group	12	6.80	0.07	6.79	6.74	6.95	6.74	6.84	*p* < 0.001 *
	Control group	8	4.02	0.13	3.98	3.89	4.21	3.94	4.06	
P3	Study group	12	6.46	0.03	6.45	6.45	6.53	6.45	6.46	*p* < 0.001 *
	Control group	8	4.58	0.40	4.56	4.12	5.09	4.29	4.86	
Fz	Study group	12	5.90	0.06	5.93	5.75	5.93	5.91	5.93	*p* < 0.001 *
	Control group	8	4.53	0.54	4.47	3.94	5.23	4.13	4.87	
Cz	Study group	12	5.68	0.03	5.67	5.66	5.76	5.67	5.67	*p* < 0.001 *
	Control group	8	4.00	0.24	3.94	3.78	4.36	3.84	4.10	
F4	Study group	12	5.39	0.36	5.43	4.97	5.73	5.09	5.73	*p* < 0.001 *
	Control group	8	4.14	0.19	4.17	3.89	4.34	4.03	4.29	
C4	Study group	12	5.63	0.11	5.61	5.53	5.78	5.53	5.73	*p* < 0.001 *
	Control group	8	4.70	0.63	4.64	3.99	5.51	4.24	5.11	
P4	Study group	12	5.57	0.26	5.44	5.38	5.98	5.38	5.66	*p* < 0.001 *
	Control group	8	4.44	0.40	4.56	3.82	4.81	4.38	4.62	

*p*—Mann–Whitney test, SD—standard deviation, Q1—lower quartile, Q3—upper quartile. * statistically significant (*p* < 0.05).

**Table 6:** The results of the alpha waves examination (waves from Fz, F3, F4, Cz, C3, C4, P3, and P4 channels). The values express wave amplitude (in µV) with main distribution parameters (including median and quartiles).

Point	Group	N	Mean	SD	Median	Min	Max	Q1	Q3	*p*
F3	Study group	12	12.32	1.03	12.72	10.56	13.76	11.68	12.79	*p* < 0.001 *
	Control group	8	5.44	0.30	5.46	5.07	6.02	5.34	5.50	
C3	Study group	12	11.30	0.97	10.89	9.75	12.97	10.79	11.79	*p* < 0.001 *
	Control group	8	5.34	0.41	5.22	4.98	5.93	5.00	5.56	
P3	Study group	12	11.00	0.93	10.84	9.75	12.85	10.45	11.26	*p* < 0.001 *
	Control group	8	7.64	0.25	7.69	7.28	7.93	7.56	7.77	
Fz	Study group	12	11.89	1.02	11.70	10.69	13.74	10.87	12.75	*p* < 0.001 *
	Control group	8	5.28	0.49	5.02	5.02	6.13	5.02	5.27	
Cz	Study group	12	11.39	1.37	10.74	9.69	13.74	10.53	12.00	*p* < 0.001 *
	Control group	8	5.72	0.70	5.43	5.43	7.43	5.43	5.50	
F4	Study group	12	12.20	0.93	12.16	10.70	13.74	11.74	12.92	*p* < 0.001 *
	Control group	8	5.85	0.24	5.93	5.26	5.93	5.93	5.93	
C4	Study group	12	11.65	0.90	11.53	10.69	13.74	10.96	11.92	*p* < 0.001 *
	Control group	8	6.06	0.19	6.13	5.58	6.13	6.13	6.13	
P4	Study group	12	12.47	0.77	12.70	10.69	13.74	12.20	12.79	*p* < 0.001 *
	Control group	8	7.57	0.28	7.50	7.29	7.99	7.41	7.67	

*p*—Mann–Whitney test, SD—standard deviation, Q1—lower quartile, Q3—upper quartile. * statistically significant (*p* < 0.05).

## Data Availability

All data are included in the manuscript.
